# Highly Sensitive Electrochemical Non-Enzymatic Uric Acid Sensor Based on Cobalt Oxide Puffy Balls-like Nanostructure

**DOI:** 10.3390/bios13030375

**Published:** 2023-03-12

**Authors:** Vandana Nagal, Sakeena Masrat, Marya Khan, Shamshad Alam, Akil Ahmad, Mohammed B. Alshammari, Kiesar Sideeq Bhat, Sergey M. Novikov, Prabhash Mishra, Ajit Khosla, Rafiq Ahmad

**Affiliations:** 1 Centre for Nanoscience and Nanotechnology, Jamia Millia Islamia, New Delhi 110025, India; 2 Department of Pharmacology & Therapeutics, Rosewell Park Cancer Institute, Elm Street & Carlton Street, Buffalo, NY 14263, USA; 3 Department of Chemistry, College of Science and Humanities in Al-Kharj, Prince Sattam Bin Abdulaziz University, Al-Kharj 11942, Saudi Arabia; 4 Department of Bioresources, University of Kashmir, Hazratbal, Srinagar 190006, India; 5 Singapore-MIT Alliance for Research and Technology (SMART), Critical Analytics for Manufacturing Personalized-Medicine (CAMP), Create Way 138602, Singapore; 6 Center for Photonics and 2D Materials, Moscow Institute of Physics and Technology, Dolgoprudny 141700, Russia; 7 Department of Applied Chemistry, School of Advanced Materials and Nanotechnology, Xidian University, Xi’an 710126, China

**Keywords:** cobalt oxide, puffy balls nanostructure, cyclic voltammetry, high sensitivity, uric acid, differential pulse voltammetry

## Abstract

Early-stage uric acid (UA) abnormality detection is crucial for a healthy human. With the evolution of nanoscience, metal oxide nanostructure-based sensors have become a potential candidate for health monitoring due to their low-cost, easy-to-handle, and portability. Herein, we demonstrate the synthesis of puffy balls-like cobalt oxide nanostructure using a hydrothermal method and utilize them to modify the working electrode for non-enzymatic electrochemical sensor fabrication. The non-enzymatic electrochemical sensor was utilized for UA determination using cyclic voltammetry (CV) and differential pulse voltammetry (DPV). The puffy balls-shaped cobalt oxide nanostructure-modified glassy carbon (GC) electrode exhibited excellent electro-catalytic activity during UA detection. Interestingly, when we compared the sensitivity of non-enzymatic electrochemical UA sensors, the DPV technique resulted in high sensitivity (2158 µA/mM.cm^2^) compared to the CV technique (sensitivity = 307 µA/mM.cm^2^). The developed non-enzymatic electrochemical UA sensor showed good selectivity, stability, reproducibility, and applicability in the human serum. Moreover, this study indicates that the puffy balls-shaped cobalt oxide nanostructure can be utilized as electrode material for designing (bio)sensors to detect a specific analyte.

## 1. Introduction

Uric acid (UA) is a waste product in human fluids (i.e., blood and urine). It is produced when the chemical known as purine nucleotides (natural substances present in the body) is broken down by the body. The normal ranges of uric acid in urine and serum for healthy individuals are 1.40–4.40 mM and 0.30–0.50 mM, respectively [1]. The abnormal presence of UA concentration in the body is harmful [2,3,4]. When the UA concentration in the blood increases from the normal level, it causes diseases like gout and other related ailments, such as tumor lysis syndrome, diabetes, uric acid stone formation, and many more. On the other hand, a decrease in UA concentration from the normal level leads to hypouricemia, which in turn leads to chronic kidney failure, multiple sclerosis, etc. [5,6,7]. Therefore, early diagnosis of abnormal UA levels is necessary to prevent certain health issues.

Several standard methods are available for UA detection, like mass spectrometry/chromatography, electrochemical enzymatic/non-enzymatic technique, and phosphotungstic acid reduction [8,9,10,11,12]. Mass spectrometry/chromatography is a suitable choice because of its high sensitivity and good reliability, but the major drawback of this method is the high cost of the instrument. The reduction of phosphotungstic acid is a simple and easy approach compared to other methods, but its operation is restricted due to interference effects. Therefore, electrochemical sensing approaches are preferred over other methods in analytical chemistry for the accurate quantitative estimation of parameters like sensitivity, selectivity, and responsivity. In addition, these electrochemical sensors offer small size, minimum cost, long-term sustainability/storage, reliability, and eco-friendliness as important factors [13,14,15]. On the other hand, the electrochemical enzyme-based sensors are highly specific but not a good choice because of their complicated enzyme immobilization techniques, high enzyme costs, low repeatability rate, long-term storage/stability issues, and severe operating conditions [16,17,18]. Therefore, the researcher’s focus has switched towards non-enzymatic electrochemical sensing devices that utilize special properties of metal/metal oxide nanostructures [19,20,21]. The non-enzymatic sensors, when fabricated with desired metal/metal oxide nanostructures, show exceptional capability and sensing performance for detecting analytes like glucose, ascorbic acid, dopamine, hydrazine, uric acid, etc. [22,23,24,25,26,27,28].

The metal oxide nanomaterials (e.g., zinc oxide (ZnO), nickel oxide (NiO_x_), manganese oxide (Mg_2_O), zirconium oxide (Zr_2_O), iron oxide (Fe_2_O_3_), and cobalt oxide (Co_3_O_4_))- based electrochemical sensors are catching the interest of researchers due to their extraordinary physical properties like reliability, stability, small packaging, and minimum cost [29,30,31,32,33,34]. Nanostructures of cobalt oxide are being used to construct non-enzymatic electrochemical sensors due to simple and low-cost synthesis processes, chemical stability, and fast charge transfer property [29,30,35]. Recently, nano-berry, porous, and hexagonal nanosheet-like nanostructures of cobalt oxides were utilized to fabricate non-enzymatic UA sensors [36,37,38]. Kogularasu et al. synthesized polyhedron-shaped Co_3_O_4_ nanostructures to construct a high-sensitivity hydrogen peroxide sensor [39]. Zhang et al. described the effect of Co_3_O_4_ nanosheets on enzymeless glucose detection [40]. Kang et al. investigated the effect of nanowire shapes like Co_3_O_4_ nanostructures on the glucose sensor [41]. Mondal et al. reported an enzymeless glucose sensor by utilizing various nanostructures of Co_3_O_4_ (i.e., nanoflowers, porous nanorods, and spherical nanoparticles) [42]. Chang et al. employed several structures of Co_3_O_4_, for example, nanowire, lump, and flower-like morphologies, to detect lactic acid [43]. However, till now, there are no reports on puffy balls-shaped Co_3_O_4_ nanostructures based on non-enzymatic electrochemical UA sensing.

In this work, we designed a non-enzymatic electrochemical UA sensor using puffy balls-like cobalt oxide nanostructure. First, puffy balls-like cobalt oxide nanostructures were synthesized using a low-cost hydrothermal method and characterized with different techniques for structural and morphological analysis. Then, the non-enzymatic electrochemical UA sensor (cobalt oxide puffy balls/GCE) was fabricated, and the sensing activity of the as-prepared nanostructured-based sensor was investigated using electrochemical techniques (i.e., CV, electrochemical impedance spectroscopy (EIS), and DPV). The cobalt oxide puffy balls/GCE sensor using the DPV technique exhibited excellent sensitivity (2158 µA/mM.cm^2^) towards UA detection. In addition, selectivity, stability, reproducibility, and UA detection in human serum were conducted to establish the feasibility of the sensor for future applications.

## 2. Materials and Methods

### 2.1. Chemicals

Cobalt nitrate hexahydrate (purity ≥ 99.99%, Co(NO_3_)_2_.6H_2_O), urea (purity 99%), ethanol (laboratory reagent), 2-(2-Butoxyethoxy) ethyl acetate (purity ≥ 99.2%, binder), uric acid (purity ≥ 99%), potassium chloride (KCl), sodium chloride (NaCl), potassium hexacyanoferrate (purity ≥ 99%, K_3_[Fe(CN)_6_]), and phosphate buffer saline (pH = 7.4, PBS) solution were purchased from Sigma-Aldrich. All analytical grade chemicals were used for this work.

### 2.2. Synthesis of Puffy Balls-like Cobalt Oxide Nanostructures

A low-cost and low-temperature hydrothermal method has been utilized for the growth of puffy balls-shaped cobalt oxide nanostructures. Precisely, 1.45 g of cobalt nitrate hexahydrate and urea were dissolved separately in 20 mL of DI water. Both prepared solutions were thoroughly mixed (total 40 mL) using magnetic stirring for 10 min at 450 rpm. The as-prepared homogeneous solution was poured into a stainless-steel lined autoclave and maintained at 150 °C for 4 h. When the autoclave was cooled to room temperature, the black solution was washed using ethanol and DI water to remove the impurities. After washing, the black precipitate sample was vacuum dried at 60 °C for 24 h. In the end, the black powder sample was vacuum annealed at 500 °C for 3 h at a ramp rate of 10 °C/minute before characterization.

### 2.3. Non-Enzymatic Electrochemical UA Sensor Fabrication

**Figure 1:**
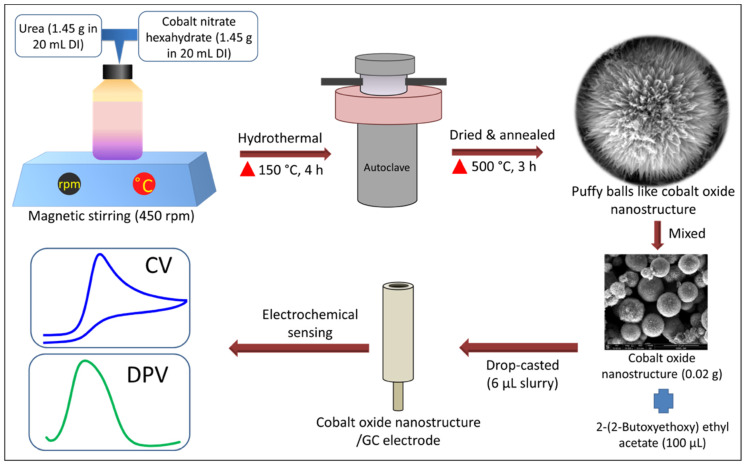
Schematic representation of puffy balls-like cobalt oxide nanostructures synthesis and non-enzymatic electrochemical UA sensor fabrication process.

To fabricate a non-enzymatic electrochemical UA sensor, the GC electrode was first polished and then cleaned using ultrasonication several times in nitric acid, ethanol, and DI water. Then, a slurry of puffy balls-shaped cobalt oxide nanostructure (0.02 g) was prepared in 100 µL binder (2-(2-Butoxyethoxy) ethyl acetate) with the help of ultrasonication for 10 min to get a uniform suspension. Further, an optimized 6 µL slurry was drop-casted onto the working GC electrode and kept for drying in the oven for 6 h. A detailed sensor fabrication procedure is shown in Figure 1.

### 2.4. Material Characterization and Electrochemical Sensing Analysis Equipment

The field-emission-scanning-electron-microscope (FESEM; Zeiss, Oberkochen, Germany) was used to characterize the morphology of the puffy balls-shaped cobalt oxide nanostructure. The crystal structure and phase purity integrity were characterized using an X-ray diffractometer (XRD; Rigaku). XRD machine was equipped with Cu-K_α_ radiation source of wavelength (1.5418 Å), operating voltage (40 kV), and current (30 mA). The ASAP 2010 analyzer was utilized for BET (Brunauer–Emmett–Teller) analysis at 77 K. The UA electrochemical sensing ability of fabricated cobalt oxide puffy balls/GC non-enzymatic electrochemical UA sensor was tested via a portable potentiostat “Palmsense4” connected with a conventional 3-electrode (working (GC), counter (Pt wire), reference (Ag/AgCl, 3M KCl)) electrochemical workstation. The electrochemical analysis was performed using EIS, CV, and DPV measurements. We utilized the previously optimized cobalt oxide amount and pH to get the optimum sensing response of the fabricated sensor [36,37,38]. The CV was performed in a 5 mM probe solution of potassium hexacyanoferrate (purity ≥ 9 9%, K_3_[Fe(CN)_6_]) in 0.1 M KCl under varying scan rates from 10 mVs^−1^ to 200 mVs^−1^. The EIS was studied in the frequency range of 0.1 to 10^−5^ Hz. The UA analyte sensing was performed using CV (voltage range = −0.20 V to +0.80 V) and DPV (voltage range = +0.20 V to +0.70 V).

## 3. Results

### 3.1. Analysis of Cobalt Oxide Nanostructure

**Figure 2:**
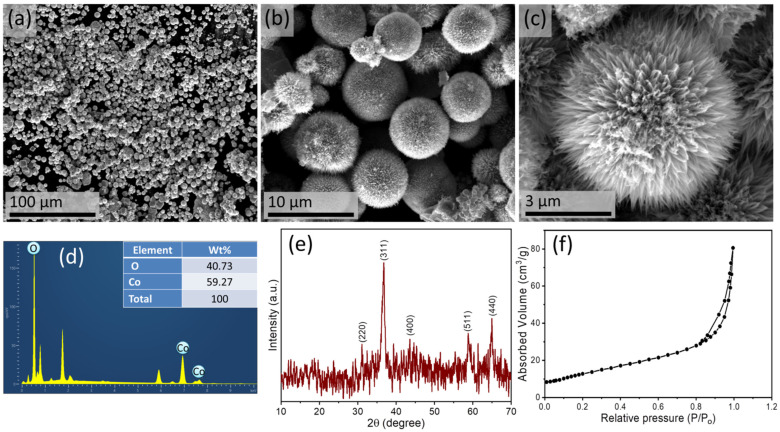
FESEM images at different magnifications (**a**–**c**), EDX analysis (**d**), XRD pattern (**e**), and BET analysis (**f**) of puffy balls-like cobalt oxide nanostructure.

The morphology of synthesized cobalt oxide nanostructures is shown in FESEM images (Figure 2a–c), which depicts the successful formation of puffy balls-like cobalt oxide nanostructures. These nanostructures are synthesized in uniform shape, evident from the low-magnification FESEM image, shown in Figure 2a. The high-resolution FESEM image shows many pine needle-like nanostructures joined at the top to form a chrysanthemum-like hierarchical structure (Figure 2c). The energy-dispersive X-ray (EDX) analysis confirms this.

The composition of puffy balls-like cobalt oxide nanostructure (Figure 2d). The EDX shows only two elements (i.e., oxygen and cobalt). Further, the XRD pattern was investigated to study the crystallinity and crystal phase of puffy balls-like cobalt oxide nanostructures, as shown in Figure 2e. The synthesized crystal structure and obtained diffraction peaks are in good agreement with standard JCPDS card# 42-1467 [39,42]. The different diffraction (2θ values) planes with corresponding miller indices (220), (311), (4000), (511), and (440) were obtained. A clear peak for the (311) plane is of the highest intensity, indicating the crystalline nature of synthesized cobalt oxide nanostructures [44]. Additionally, we analyzed the surface area of the cobalt oxide puffy balls-like nanostructure using BET analysis (Figure 2f). The specific surface area was ~93 m^2^/g, which suggests cobalt oxide puffy balls-like nanostructures are suitable nanomaterials as high-performance catalysts.

### 3.2. Electrochemical Sensing Analysis of Cobalt Oxide Puffy Balls/GC Electrodes

**Figure 3:**
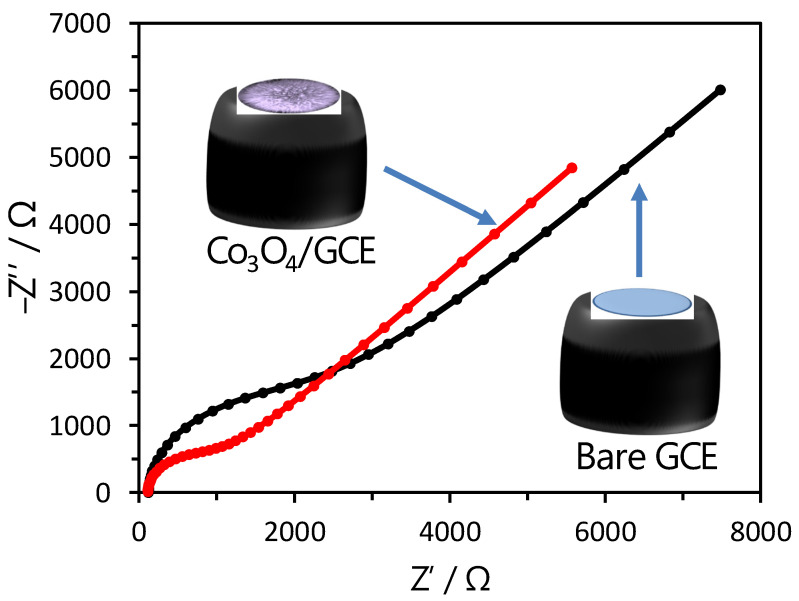
Nyquist plots of bare GC and cobalt oxide puffy balls/GC sensors in probe solution (5 mM, K_3_[Fe(CN)_6_] and 0.1 M KCl); frequency range = 0.1 Hz to 10^−5^ Hz. Insets show the schemes of bare (above inset) and modified (below inset) GC electrodes.

The EIS and CV studies were performed in the potassium hexacyanoferrate (K_3_[Fe(CN)_6_]; 5 mM) and 0.1 M KCl solution to investigate the electron transfer capability of the puffy balls-shaped cobalt oxide nanostructure-modified sensor electrode. The Nyquist plots of obtained EIS spectra profile for bare GC and cobalt oxide puffy balls/GC electrodes (Figure 3). The Nyquist plot has a semicircle in a high-frequency region corresponding to the charge transfer-controlled process and a straight line corresponding to the mass transfer diffusion-controlled process. The charge transfer resistance value was determined by the semicircle diameter intercept value on the x-axis. The calculated charge transfer resistance for bare GC electrode (~2230 Ω) is higher compared to cobalt oxide puffy balls/GC electrode (~980 Ω), indicating better charge transfer capabilities of cobalt oxide puffy balls nanostructures.

**Figure 4:**
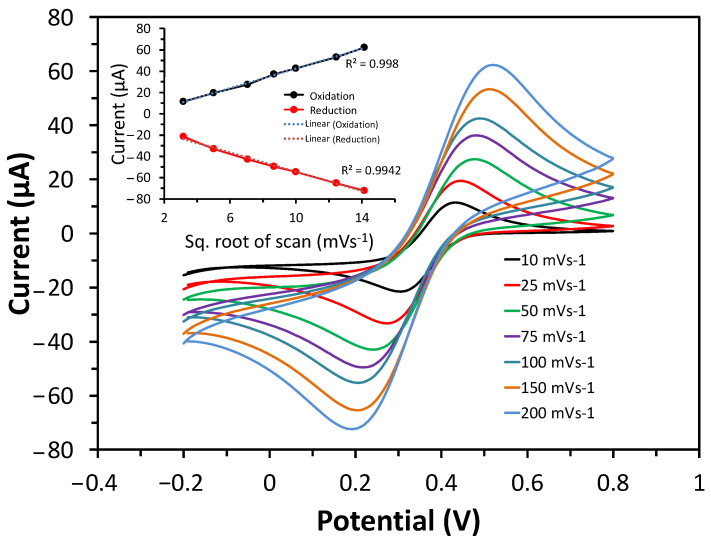
CV curve of cobalt oxide puffy balls nanostructures/GC electrode measured in probe solution from 10 to 200 mVs^−1^ scan rates. Inset shows the anodic/cathodic current vs. square root of the scan rate plot.

To further validate the electron transfer of cobalt oxide puffy balls/GC electrodes, the CV response was measured at varying scan rates (10 to 200 mVs^−1^) (Figure 4). The CV response shows the increase in peak current with an increasing scan rate. This increase is linear when drawing a plot for anodic/cathodic current vs. square root of scan rate plot (inset of Figure 4). The cobalt oxide puffy balls/GC electrodes showed a typical diffusion-controlled process for the reversible system [36]. Both CV and EIS measurements in the probe solution confirm the fast electron transfer capability of cobalt oxide puffy balls/GC electrode.

### 3.3. Detection of UA Using CV

**Figure 5:**
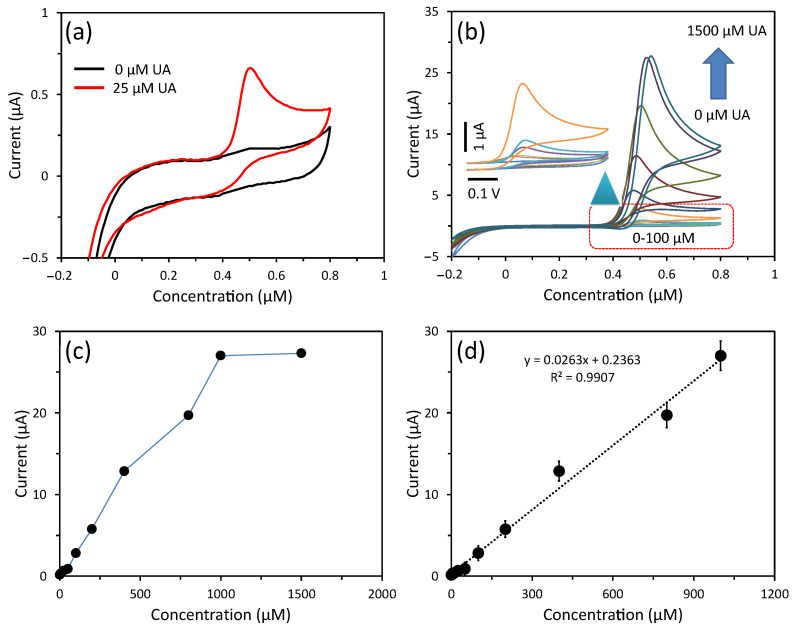
(**a**) CV response of cobalt oxide puffy balls/GC sensor towards 0 μM and 25 μM UA concentration and (**b**) CV response for 0 to 1500 μM UA concentration in PBS buffer (pH = 7.4). (**c**) current response vs. UA concentration plot, and (**d**) calibrated plot showing slope (0.0263 μA/μM) and regression coefficient (R^2^) of 0.9907. Inset b presents CV response at a low-concentration range (0 to 100 μM) of UA.

To investigate the electrochemical non-enzymatic sensing of fabricated cobalt oxide puffy balls/GC sensor, the CV response was measured in PBS buffer solution (pH = 7.4) having UA at 50 mVs^−1^ scan rate. Figure 5a depicts the CV response curve of the cobalt oxide puffy balls/GC sensor for 0 mM and 25 mM UA concentrations. A sharp increase in current response is seen in the presence of UA due to the UA oxidation, where the p-type nature of the cobalt oxide semiconductor plays a crucial role in swift electron transfer to the electrode surface by providing excess hole concentration [30,36,37]. A detailed UA sensing mechanism over cobalt oxide is proposed by Hu et al. [45]. Furthermore, a detailed study of the sensor performance was conducted by measuring UA concentrations from 0 µM to 1500 µM in PBS buffer solution (Figure 5b). With increasing UA concentration in the buffer solution, the non-enzymatic cobalt oxide puffy balls/GC sensor showed an enhanced current response. The peak current vs. UA concentration plot is plotted in Figure 5c, which shows a linear current increase up to 1000 µM of UA. Further increase in UA concentration shows a non-linear curve due to saturation of the cobalt oxide puffy balls/GC sensor surface. Similarly, we repeated these electrochemical non-enzymatic sensing experiments three times, and their calibration plot of the linear range is plotted in Figure 5d. The calibration plot shows the slope of 0.0263 µA/µM with high regression coefficient (R^2^) of 0.9907. The fabricated cobalt oxide puffy balls/GC sensor exhibited a sensitivity of 370 µA/mM.cm^2^. Additionally, the limit of detection was calculated to be 2.4 µM based on the signal and noise ratio of 3. The sensing performance of the cobalt oxide puffy balls/GC sensor is compared in Table 1. The high sensitivity is due to the large surface area to volume ratio and p-type semiconducting nature of puffy balls-like cobalt oxide nanostructures [36,37].

### 3.4. Detection of UA Using DPV

DPV is a pulsed technique in which ramp voltage is applied. Due to the short sampling time of applied ramp voltage in DPV, the capacitive charging current is minimized compared to the Faradaic current, and the ratio of Faradaic current to non-Faradaic current increases, which makes DPV more sensitive compared to the CV technique [32]. In addition, DPV accurately accounts for double-layer capacitance in which changes occur even when a small molecule of analyte absorbs at the electrode surface with pulse sampling. In contrast, CV suffers from excessive capacitance changes [33,46]. Therefore, we utilized the DPV technique to detect UA.

**Figure 6:**
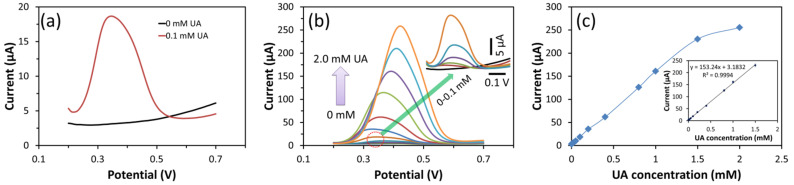
DPV response of cobalt oxide puffy balls/GC sensor towards 0 mM and 0.1 mM UA (**a**), from 0 to 2.0 mM UA in PBS buffer (pH = 7.4) (**b**), and current response vs. UA concentration plot (**c**). Inset b is the DPV response at a low-concentration range (0 to 0.1 mM) of UA. Inset c represents the calibrated plot showing a slope (153.24 μA/mM) and R^2^ of 0.9994.

Figure 6 depicts the measured DPV and calibration curves for different UA concentrations (0 mM to 2 mM) in the 0.2 V to 0.7 V voltage range. The cobalt oxide puffy balls/GC sensor responded in the presence of UA in the buffer due to UA oxidation (Figure 6a). In addition, the DPV oxidation current increased with UA concentration (Figure 6b).

A linear increase in current is observed with increasing UA concentration up to 1.5 mM (Figure 6c). Further from the obtained calibration plot, the cobalt oxide puffy balls/GC sensor resulted in high sensitivity of 2158 µA/mM.cm^2^, which is ~6 times more sensitive towards UA compared to the CV-based sensitivity of cobalt oxide puffy balls/GC sensor (Table 1). This high sensitivity of the fabricated sensor is attributed to nanostructure morphology that offers more active sites available at the surface of puffy balls and the p-type semiconduction nature of cobalt oxide.

### 3.5. Selectivity, Stability, and Reproducibility Tests

**Figure 7:**
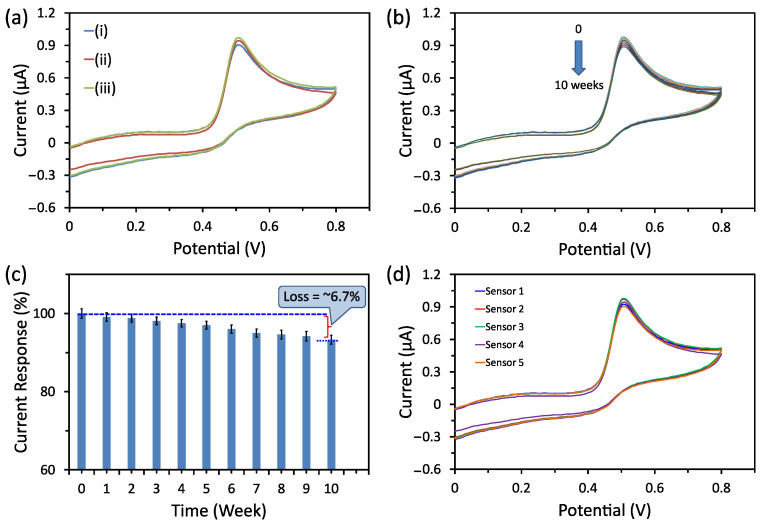
(**a**) CV scans of cobalt oxide puffy balls/GC sensor in PBS buffer containing (i) 50 µM UA only, (ii) 50 µM UA and 50 µM of each possible interfering species (i.e., urea, sodium chloride, fructose, glucose, potassium chloride, L-cysteine, and lactic acid), and (iii) 50 µM UA and 100 µM of each above-interfering species. (**b**) Sensing response of cobalt oxide puffy balls/GC sensor towards 50 µM UA for 10 weeks storage time. (**c**) Calibrated response of the sensor showing ~6.7% decrease compared to the sensor fabricated on day 1. (**d**) CV scans of five similar fabricated cobalt oxide puffy balls/GC sensors in PBS buffer containing 50 µM UA.

Selectivity, long-term stability, and fabrication reproducibility are significant factors for any sensor. To investigate the selectivity of the electrochemical non-enzymatic cobalt oxide puffy balls/GC sensor, the CV scans were recorded in buffer containing (i) 50 µM UA only, (ii) 50 µM UA, and 50 µM of each possible interfering species (i.e., urea, sodium chloride, fructose, glucose, potassium chloride, L-cysteine, and lactic acid), and (iii) 50 µM UA, and 100 µM of each above-interfering species (Figure 7a). The sensor showed a slightly positive CV current response in interfering species’ presence at high concentrations. Additionally, the long-term stability of the cobalt oxide puffy balls/GC sensor was evaluated for 10 weeks (Figure 7b). From the obtained CV response curves (Figure 7b), the calibrated histogram shows that the sensor holds its initial response of ~93.3%, which confirms the high storage stability of the sensors (Figure 7c). After checking the response towards UA, the sensor was rinsed in PBS buffer, dried, and kept in a desiccator.

To prevent possible fouling of the electrode. Finally, we checked the fabrication reproducibility of the cobalt oxide puffy balls/GC sensors by fabricating five sensors using the same fabrication protocols. The CV response of all five cobalt oxide puffy balls/GC sensors was tested in the PBS buffer containing 50 µM UA (Figure 7d). A low relative standard deviation (RSD) of 3.6% indicates good fabrication reproducibility.

### 3.6. UA Detection in Serum Sample

To evaluate the applicability of the fabricated cobalt oxide puffy balls/GC sensor for UA detection in serum samples (H4522; Sigma-Aldrich, Saint Louis, MO, USA), we employed a standard addition-based method. First, a known concentration of UA (i.e., 50, 100, and 200 µM) was added to the serum sample, and total UA concentration was estimated using cobalt oxide puffy balls/GC sensor. From the measurement results, recovery (%) and RSD (%) were estimated (Table 2). The results indicate good recovery and low RSD, which confirms the suitability of the cobalt oxide puffy balls/GC sensor for detecting UA in real samples.

## 4. Conclusions

In summary, we synthesized puffy balls-like cobalt oxide nanostructures using the hydrothermal method and characterized their crystal structure and surface morphology using different techniques (i.e., XRD and FESEM). The non-enzymatic electrochemical UA sensor was designed with as-synthesized puffy balls-like cobalt oxide nanostructures. The sensing performance of fabricated cobalt oxide puffy balls/GC sensor was tested for UA detection using two techniques (i.e., CV and DPV), and their sensing performance was compared. The fabricated cobalt oxide puffy balls/GC sensor exhibited high sensitivity towards UA, which is ~6 times more sensitive when measured using the DPV technique (2158 µA/mM.cm^2^) compared to the sensitivity obtained with CV (370 µA/mM.cm^2^). Additionally, the cobalt oxide puffy balls/GC sensor showed good selectivity, long-term stability, fabrication reproducibility, and applicability in the human serum sample. The puffy balls-shaped cobalt oxide nanostructure with a high surface area can be an exciting working electrode nanomaterial for fabricating different sensors using a specific enzyme/other metal or metal oxide modifications.

## Figures and Tables

**Table 1:** Comparison of electrochemical non-enzymatic UA sensing performance of cobalt oxide puffy balls/GC sensor with previously published literature.

Modified Electrode	Sensing Method	Detection Range (µM)	Limit of Detection (µM)	Sensitivity (µAcm^−2^mM^−1^)	Ref.
g-C_3_N_4_ NSs/GCE	DPV	100–1000	4.45	-	[7]
Ag-Fe_2_O_3_@PANI	DPV	0.001–0.90	0.000102	128290	[21]
Au/RGO/GCE	DPV	8.8–53	1.8	-	[22]
Cubic Pd/RGO/GCE	DPV	4–469.5	1.6	-	[23]
RGO/PB 100/GCE	CV	40–415	8.0	-	[24]
PCN/MWCNT/GCE	DPV	0.2–20	0.139	-	[25]
B-MWCNT/GCE	CV	60–250	0.65	-	[26]
PtNi@MoS_2_ NSs/GCE	DPV	0.5–600	0.1	-	[27]
Cysteic acid/GCE	DPV	1.0–19	0.36	-	[28]
PPy-CB-Co_3_O_4_/GCE	CV	0.75–305	0.46	0.8786	[30]
Cu_2_O/ferrocene/uricase/GCE	DPV	0.1–1000	0.0596	1.9	[33]
Co_3_O_4_ nano berries/GCE	CV	5–3000	2.4	206	[36]
Co_3_O_4_ porous NSs/GCE	CV	0–2500	10	470	[37]
Silky Co_3_O_4_ nanomaterial/GCE	CV	500–3500	100	-	[46]
MnO_2_ NFs/NG/GCE	SDLSV	10–100	0.039	-	[47]
Co_3_O_4_ puffy balls/GCE	CV	0–1000	2.4	307	This work
Co_3_O_4_ puffy balls/GCE	DPV	0–1500	1.6	2158	This work

Abbreviations: Graphitic carbon nitride—g-C_3_N_4_; nanosheets—NSs; glassy carbon electrode—GCE; gold—Au; differential pulse voltammetry—DPV; silver—Ag; iron oxide—Fe_2_O_3_; polyaniline—PANI; reduced graphene oxide—RGO; palladium—Pd; Prussian blue—PB; cyclic voltammetry—CV; porous carbon nitride—PCN; multi-wall carbon nanotube—MWCNT; platinum—Pt; nickel—Ni; molybdenum disulfide—MoS_2_; polypyrrole—Ppy; carbon black—CB; cobalt oxide—Co_3_O_4_; cuprous oxide—Cu_2_O; manganese dioxide—MnO_2_; nanoflowers—NFs; nitrogen-doped graphene—NG; second-derivative linear sweep voltammetry—SDLSV.

**Table 2:** UA analysis in human serum (H4522) sample using fabricated cobalt oxide puffy balls/GC sensor.

Sample	Added UA (µM)	Found UA (µM)	Recovery (%)	RSD (%) (*n* = 3)
Human serum (H4522)	0	280	-	-
	50	328.7	97.4	3.1
	100	196.6	96.6	2.8
	200	391.6	95.8	3.4

## Data Availability

Not applicable.
